# Gene Level Regulation of Na,K-ATPase in the Renal Proximal Tubule Is Controlled by Two Independent but Interacting Regulatory Mechanisms Involving Salt Inducible Kinase 1 and CREB-Regulated Transcriptional Coactivators

**DOI:** 10.3390/ijms19072086

**Published:** 2018-07-18

**Authors:** Mary Taub

**Affiliations:** Biochemistry Dept., Jacobs School of Medicine and Biomedical Sciences, University at Buffalo, 955 Main Street, Suite 4902, Buffalo, NY 14203, USA; biochtau@buffalo.edu; Tel.: +1716-829-3300; Fax: +1716-829-2132

**Keywords:** Na,K-ATPase, kidney, proximal tubule, renal cell culture, catecholamines, CREB, CRTC, SIK1, Histone Deacetylase

## Abstract

For many years, studies concerning the regulation of Na,K-ATPase were restricted to acute regulatory mechanisms, which affected the phosphorylation of Na,K-ATPase, and thus its retention on the plasma membrane. However, in recent years, this focus has changed. Na,K-ATPase has been established as a signal transducer, which becomes part of a signaling complex as a consequence of ouabain binding. Na,K-ATPase within this signaling complex is localized in caveolae, where Na,K-ATPase has also been observed to regulate Inositol 1,4,5-Trisphosphate Receptor (IP3R)-mediated calcium release. This latter association has been implicated as playing a role in signaling by G Protein Coupled Receptors (GPCRs). Here, the consequences of signaling by renal effectors that act via such GPCRs are reviewed, including their regulatory effects on Na,K-ATPase gene expression in the renal proximal tubule (RPT). Two major types of gene regulation entail signaling by Salt Inducible Kinase 1 (SIK1). On one hand, SIK1 acts so as to block signaling via cAMP Response Element (CRE) Binding Protein (CREB) Regulated Transcriptional Coactivators (CRTCs) and on the other hand, SIK1 acts so as to stimulate signaling via the Myocyte Enhancer Factor 2 (MEF2)/nuclear factor of activated T cell (NFAT) regulated genes. Ultimate consequences of these pathways include regulatory effects which alter the rate of transcription of the Na,K-ATPase β1 subunit gene *atp1b1* by CREB, as well as by MEF2/NFAT.

## 1. Introduction

During the last 20 years, Na,K-ATPase has been established as a signal transducer, in addition to its role in transport [1]. Na,K-ATPase undergoes an E1 to E2 conformational change coupled to the movement of ions across the plasma membrane [2]. For many years, it has been known that Na,K-ATPase is a highly specific receptor for ouabain and other cardiotonic steroids. The binding of ouabain to Na,K-ATPase keeps Na,K-ATPase in an E2-like closed conformation, so as to inhibit the ion pumping activity of the enzyme [3]. This attribute of ouabain has led to its use in treating heart failure. Ouabain at concentrations non-inhibitory to Na,K-ATPase activity can also stimulate cell growth [4]. Xie and coworkers demonstrated that ouabain binding at low concentrations stimulates the formation of a signaling complex containing the Na,K-ATPase, Src, and the EGF Receptor (EGFR) [5]. Signaling pathways which emerge include Mitogen Activated Protein Kinase (MAPK), Ca^2+^, and Reactive Oxygen Species (ROS), with downstream consequences on gene expression. Initial studies concerning ouabain and signaling were conducted with cardiomyocytes. This report is concerned with Na,K-ATPase signaling by the CRTC/SIK1 pathway in the kidney, specifically the RPT [6].

The RPT reabsorbs the majority of the Na^+^ in the glomerular filtrate. However, the actual quantity of Na^+^ reabsorption by the RPT depends upon dietary Na^+^ levels, as well as the control mechanisms in place in this nephron segment [7,8]. There is growing evidence that the RPT plays a major role in blood pressure regulation, primarily from studies with animal models. Examples include the hypotension in an RPT specific Angiotensin II receptor (AT1A) knockout [9], and the salt-sensitive hypertension in a RPT specific Knockout (KO) of aromatic L-amino acid decarboxylase [10], the enzyme responsible for renal dopamine synthesis. In addition are studies with animals with mutations in dopamine and adrenergic receptors [11,12,13,14,15,16,17,18], as well as directed KOs in RPT transporters including NHE3 and NBCe2 [11,12,13,14,15,16,17,18,19,20]. All of the altered genes affect Na^+^ efflux by RPT Na,K-ATPase. 

Na,K-ATPase is essential for transepithelial transport by kidney tubule epithelial cells, in addition to its role in cell volume regulation. Na,K-ATPase plays a number of roles in these regards. First of all, the majority of the Na^+^ which enters RPT cells by apical transporters, leaves the cells via Na,K-ATPase. Secondly, Na,K-ATPase establishes the Na^+^ gradients required for the reabsorption of other solutes by Na^+^/solute cotransporters, including amino acids, sugars, bicarbonate, phosphate, and lactate in the RPT [21]. Thirdly, Na,K-ATPase plays an essential regulatory role in signal transduction. In addition to activating signaling transduction pathways, Na,K-ATPase itself is also subject to control by signal transduction pathways. 

## 2. Response of the RPT to Changes in Na^+^ Intake

The RPT is of particular interest, responding in a bidirectional manner to changes in Na^+^ intake. When luminal Na^+^ levels decline, renal effectors are produced, which activate signaling pathways promoting Na^+^ conservation. In contrast, when luminal Na^+^ levels increase, other renal effectors are produced, which activate signaling pathways promoting Na^+^ excretion. The regulatory mechanisms which are activated in response to changes in luminal Na^+^ often involve “natritropic” nerves and hormones, and are distinct from the mechanisms responsible for maintaining the glomerulotubular balance [22]. The RPT is the major nephron segment in which the glomerulotubular balance operates, such that approximately 67% of the filtered load is reabsorbed regardless of the glomerular filtration rate (GFR) [22]. 

Included amongst the effectors produced when luminal Na^+^ levels increase is norepinephrine, which is synthesized by renal nerves [23]. When norepinephrine interacts with α adrenergic receptors in the RPT, Na,K-ATPase is acutely activated, and Na^+^ reabsorption rapidly increases, resulting in antinatriuresis [24]. Signaling pathways that are involved are activated by an increase in intracellular Ca^2+^ ([Ca^2+^]_in_), which ultimately results in the dephosphorylation (and activation) of Na,K-ATPase by Ca^2+^/calmodulin dependent Protein Phosphatase 2B (PP2B, i.e., calcineurin) [25]. In contrast, following an excessive Na^+^ load, dopamine is produced by RPT cells, using circulating L-DOPA as a substrate [16]. Dopamine is externalized, and interacts in an autocrine manner with DA1 receptors on RPT cells, resulting in the phosphorylation (and inhibition) of Na,K-ATPase, and natriuresis [26]. The responses of the RPT to rapid and constantly changing levels of luminal Na^+^ are generally characterized by the increased endocytosis of basolateral Na,K-ATPase (in the case of dopamine, during natriuresis) [27], or by the integration of preexisting Na,K-ATPases into the basolateral membrane (in the case of norepinephrine, during antinatriuresis).

The actions of natriuretic and antinatriuretic effectors on Na,K-ATPase in the RPT have been studied in great detail following the pioneering studies of Aperia et al. [16]. Modest increases in intracellular Na^+^ ([Na^+^]_in_) that occur following an Na^+^ load have profound effects on a number of signaling pathways. For example, dopamine DA1 receptors colocalize with Na,K-ATPase following an increase in ([Na^+^]_in_) in the opossum kidney cell line OK, which resembles RPT cells in many respects [28]. Following dopamine treatment, intracellular Ca^2+^ ([Ca^2+^]_in_) also increases in such RPT cells, resulting in Protein Kinase C ς (PKCς) activation, phosphorylation of the Na,K-ATPase α1 subunit, and endocytosis. As a consequence, Na^+^ reabsorption in the RPT is reduced, resulting in natriuresis.

A similar increase in [Ca^2+^]_in_ is observed in ouabain-treated cardiomyocytes [5]. When cardiomyocytes are treated in vitro with ouabain at concentrations that cause partial inhibition of Na,K-ATPase, early response genes are induced; the overall rate of transcription of total RNA increases, as well as the overall rate of protein synthesis, ultimately resulting in hypertrophy, and the expression of fetal genes including, in particular, α-actin [29]. Ouabain-induced hypertrophy exhibits significant differences mechanistically from other forms of hypertrophy, including, for example, phenylephrine induced hypertrophy, which involves G Protein Coupled Receptors (GPCRs). However, even in the case of ouabain-induced hypertrophy, an increase in [Ca^2+^]_in_ is not sufficient for the hypertrophy. Central to ouabain-induced hypertrophy is the protein complex that forms between ouabain-bound Na,K-ATPase, Src, and the EGFR, which results in the activation of a number of signaling pathways, including MAPK [5]. These changes are independent of signaling caused by increases in intracellular Na^+^, K^+^, and Ca^2+^.

## 3. Acute Regulation of Na,K-ATPase by Salt Inducible Kinase 1 (SIK1)

It was in this scientific milieu, that a number of investigators began to identify other proteins that associate with the Na,K-ATPase and regulate the response of RPT cells to physiologic effectors. For example, Sjöstrom et al. [30] found that in the OK cell line, Na,K-ATPase is included within a protein complex containing SIK1, Ca^2+^/Calmodulin-dependent protein kinase 1 (CaMK1), Protein Phosphatase Methylesterase 1 (PME1), and Protein Phosphatase 2A (PP2A). The PME1 present within this complex demethylates, and inactivates PP2A, thus preventing the dephosphorylation of the α1 subunit of Na,K-ATPase (and thus promoting its endocytosis). This ultimately changes, following an increase in [Ca^2+^]_in_.

The signaling activity via the Na,K-ATPase/SIK1 complex has been manipulated using monensin, a Na^+^ ionophore [30]. The elevated [Na^+^]_in_ caused by monensin has been observed to cause an increase in [Ca^2+^]_in_ (by increasing the activity of NCX1/2, an Na^+^/Ca^2+^ exchanger), with the consequent activation of CaMK1 [30]. As a consequence, CaMK1 phosphorylates and activates SIK1 (which is associated with Na,K-ATPase), resulting in (a) the phosphorylation of PME1 by SIK1, (b) the dissociation of PME1 from the Na,K-ATPase complex; and, ultimately, (c) the activation of PP2A, and dephosphorylation of Na,K-ATPase. Presumably, a proportion of Na,K-ATPases (which are dormant, and phosphorylated under basal conditions) are rapidly activated in this manner following acute changes in [Na^+^] in the lumen of the nephron.

While acute regulation of Na,K-ATPase is critical for survival (as Na^+^ intake constantly changes), long-term regulatory mechanisms are critical in determining blood pressure regulation. The increased use of salt in our modern diet is correlated with an increased frequency of hypertension, in comparison with unacculturated societies [31]. Our modern diet can cause a long-term change in renal function, such that the “normal” Na^+^ set-point for standard blood pressure is changed, resulting in hypertension [32]. In order to understand the nature of such long-term changes, studies at the gene level are imperative, including studies concerned with changes in transcriptional regulation, as well as epigenetic changes. Presumably, the level of renal effectors, including cardiotonic steroids, may also be altered in individuals with high blood pressure, contributing to changes in blood pressure regulation.

## 4. Chronic Regulation of Na,K-ATPase by CREB and other Transcription Factors

For these reasons, an understanding of the mechanisms regulating Na,K-ATPase expression in the kidney at the gene level is important. In the mammalian kidney, two distinct, differentially regulated genes, *atp1a1* and *atp1b1*, encode for the Na,K-ATPase α1 and β1 subunits [33]. After transcription of the two genes, the Na,K-ATPase α1 and β1 subunits are synthesized and assembled in the Endoplasmic Reticulum (ER), resulting in αβ heterodimer formation. This process is limited by the level of the β subunit in MDCK cells (α being present in excess) [34]. Thus, increases in β subunit availability result in substantial increases in αβ heterodimer [35], unlike the case with the α subunit. For this reason, my laboratory has examined β subunit regulation in detail.

**Figure 1:**
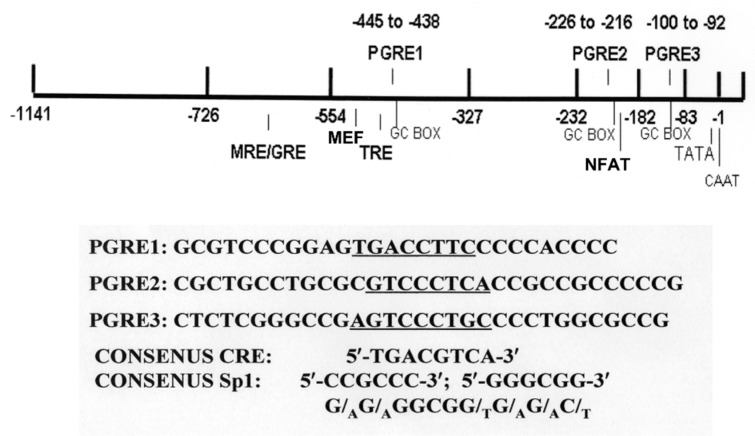
Promoter region of the human *atp1b1* gene. The human *atp1b1* promoter contains an MRE/GRE, an MEF regulatory element, a thyroid hormone regulatory element (TRE), an NFAT regulatory element, a TATA box, a CAAT box, three PGREs, and GC boxes which serve as binding sites for Sp1. The sequences of the PGREs are indicated, as well as a consensus CRE and a consensus Sp1 site (or GC box).

**Figure 2:**
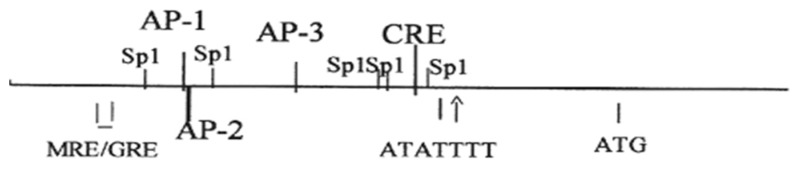
Structure of the human *atp1a1* promoter. The human *atp1a1* promoter has an MRE/GRE; AP-1, AP-2, and AP-3 sites; a CRE; and five Sp1 sites (or GC boxes).

The *atp1a1* and *atp1b1* genes are both highly conserved [17,36], having ~90% sequence identity in human, dog, rat, mouse, and rabbit specimens. The promoters also share >80% sequence identity cross-species, which (a) suggests that there are common modes of regulation; and therefore (b) validates the use of in vitro animal model systems derived from dog, rat, mouse, and rabbit specimens. Indeed, common regulatory elements are present on the *atp1a1*and *atp1b1* promoters in different species. Included amongst these regulatory elements are functional mineralocorticoid/glucocorticoid regulatory elements (MREs/GREs) located on both the *atp1a1* and *atp1b1* promoters [18,37]. A functional thyroid hormone regulatory element (TRE) has been identified on the *atp1b1* promoter by Feng et al. [38]. In addition, regulatory elements that bind the cAMP Regulatory Element Binding Protein (CREB) have been identified in the *atp1a1* promoter by Kobayashi and Kowakami [39], as well as on the *atp1b1* promoter [40,41] (Figure 1 and Figure 2) [36]. 

When considering CREB in particular, its widespread physiologic importance is indicated by studies with transgenic animals with targeted mutations in CREB in particular organs, including the brain, where neuronal excitability is affected [42]; the heart, where contractile function is impaired [43]; and the hypothalamus, which loses its control of pituitary function [44]. CREB similarly plays an essential role in glucose metabolism in the liver, spermatogenesis, and circadian rhythms [45].

Although both the *atp1a1* and the *atp1b1* promoters contain regulatory elements that bind CREB [46], the regulatory elements that recognize CREB in the promoters of these two different genes have quite different sequences. In the *atp1a1* promoter, CREB binds to a classic cAMP Regulatory Element (CRE) [39,47]. CREs with the consensus sequence, 5′-TGACGTCA-3′, and slight variants have been identified on the promoters of hundreds of cellular genes. Although the *atp1b1* promoter does not have a consensus CRE (or a close variant), three Prostaglandin Regulatory Elements (PGREs) have been identified in the *atp1b1* promoter, including PGRE1 and PGRE3, which serve as CREB binding sites (Figure 1) [41].

Despite the differences in sequences between the CRE in *atp1a1* and the PGREs in *atp1b1*, both the CRE located on the *atp1a1* promoter and the PGREs located on the *atp1b1* promoter share a unique mechanism of CREB binding, which differs from that of other known consensus CREs. Indeed, CREB binding to PGRE1 and PGRE3 requires a concomitant interaction with the Specificity Protein 1 (Sp1), located at an adjacent Sp1 site (Figure 1) [40,41]. Similarly, Kobayashi and Kowakami [39] observed that CREB binding to the CRE on the *atp1a1* promoter also involves Sp1. While the studies of Kobayashi and Kowakami [39] indicated that the CRE on the *atp1a1* promoter is involved in constitutive regulation of the transcription of the *atp1a1* gene, extensive evidence has been presented that PGRE1 and PGRE3 on the *atp1b1* promoter are involved in transcriptional regulation by effectors that interact with GPCRs [40,41,48,49]. Effectors such as PGE_2_, that interact with GPCRs that are coupled to either Gs or Gq (and that activate Protein Kinase A (PKA) and PKC), stimulate renal Na,K-ATPase transcription through the interactions that occur between CREB, Sp1, and CREB coactivators, including CREB Binding Protein (CBP) [40,50]. Transcriptional regulation by CREB in numerous mammalian genes is similarly activated by signaling via PKA, PKC, and CaMK, and/or such signaling pathways such as MAPK [51,52].

## 5. Regulation of CREB by Transcriptional Coactivators

**Figure 3:**
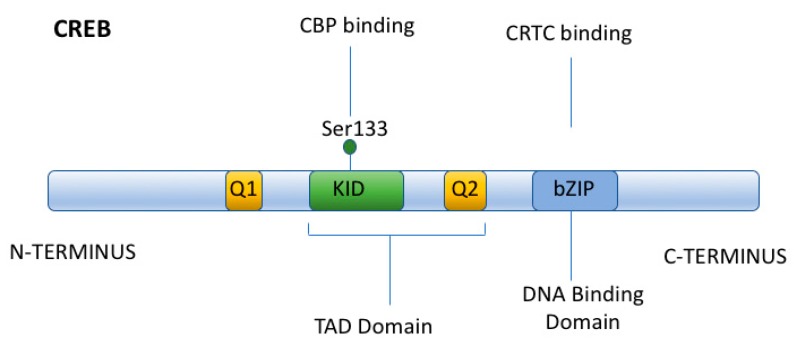
CREB Structure. CREB has two glutamic acid rich domains, Q1 and Q2; a Kinase Inducible Domain (KID); and a bZIP domain, which promotes DNA binding and CREB dimerization. PKA phosphorylates CREB at Ser133 within the KID domain, which then serves as a CBP binding site. The bZIP domain mediates CREB binding to CRTCS.

Classically, transcriptional regulation caused by increases in cAMP results in CREB phosphorylation at Ser-133 by PKA, the recruitment of CBP, and its binding to CREB (Figure 3) [53,54]. The binding of CBP to the KID domain of CREB (via CBP’s KIX domain) is generally necessary for transcriptional activation [55]. The interaction between CREB and CBP often results in the association of CBP with other transcriptional coactivators (depending upon the promoter) [56]. In addition, the association of CBP with CREB generally results in the acetylation of histones (due to the Histone Acetyltransferase (HAT) activity of CBP) [57], and in this manner opens up the local chromatin structure, so as to upregulate gene expression. This regulatory mechanism is very prevalent in the genome, with CREs being present in the promoters of approximately 5000 genes, and CREB phosphorylation at Ser-133 occurs in the majority of these genes [51].

In recent years, the classic view that CREB activation depends primarily upon its interaction with CBP has changed. Zhang et al. [58] conducted a genome wide analysis of CREB target genes in different tissues (liver, pancreas, kidney). Their analysis indicated that the activation of CREB target genes cannot always be explained simply by CREB phosphorylation. Indeed, cAMP only stimulates the transcription of about 100 CREB target genes. Admittedly, a number of the unaffected CREB target genes do not have a TATA box, a requirement for transcriptional regulation by CREB. However, when considering the genes that do have a TATA box, only 10% are transcriptionally activated by cAMP, and even a lower percentage of these genes are activated when CREB is phosphorylated at Ser-133 [51]. Thus, these investigators concluded that (a) CBP recruitment to genes containing CREB binding sites is not sufficient in many cases for transcriptional upregulation to occur (following CREB phosphorylation), and therefore (b) that additional CREB binding partners are involved in transcriptional upregulation by CREB [58]. 

One such set of alternative binding partners are CREB Regulated Transcriptional Coactivators (i.e., CRTCs, or Transducers of Regulated CREB (i.e., TORCs)) [51,59]. The CRTC family consists of three members which are present to varying extents in different mammalian tissues. CRTCs are present in numerous organisms, and their structure is evolutionarily conserved, from Drosophila melanogaster and C. elegans to man. 

**Figure 4:**
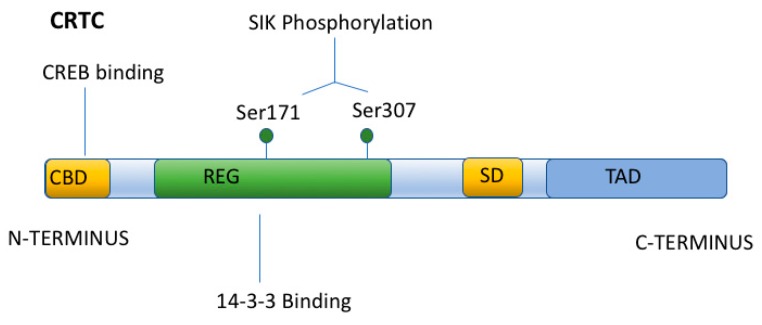
Structure of CRTC. CRTC has an amino terminal CREB binding domain (CBD), a Regulatory Domain (REG) which can be phosphorylated at two sites (Ser171 and Ser307) by SIK1, a splicing domain (SD), and a carboxy terminal Trans Activation Domain (TAD).

**Figure 5:**
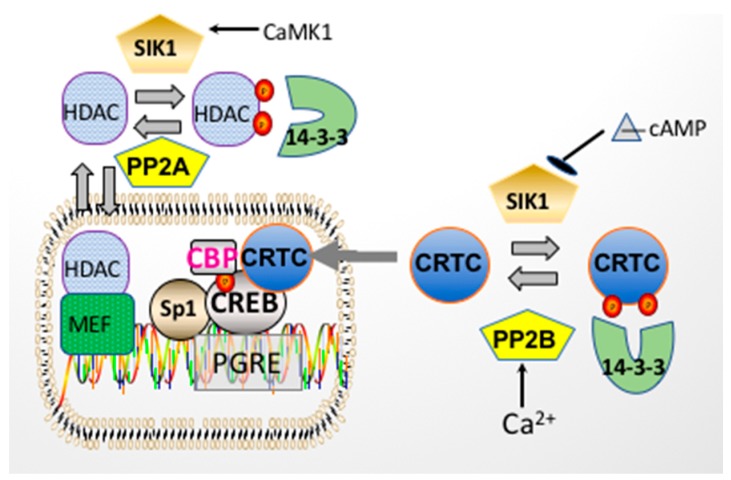
Regulation of *atp1b1* transcription by CRTCs and Class IIa HDACs. CRTCs are phosphorylated by SIK1, and translocate to the cytoplasm where they interact with 14-3-3. When SIK1 is phosphorylated and inactivated by PKA, CRTCs are then dephosphorylated by PP2B (i.e., calcineurin), provided that PP2B is activated by Ca^2+^/calmodulin. When SIK1 is phosphorylated and activated by CaMK1, SIK1 phosphorylates class IIa HDACs, which then dissociate from MEF2 transcription factors, activating transcription by MEF2. Phosphorylated class IIa HDACs translocate to the cytoplasm where they are sequestered by 14-3-3 proteins.

The general structural components of CRTC proteins include an amino terminal coil-coil CREB binding domain and a central regulatory region (subject to phosphorylation by SIK), as well as a carboxy terminal transactivation domain (or TAD) (Figure 4) [60]. Unlike CBP, which binds to the KID domain of CREB, CRTCs interact (via their amino terminal coil-coil domain) with the basic-leucine zipper (bZIP) domain of CREB (Figure 4), so as to promote DNA binding and CREB dimerization. Thus, the activation of CREB by CRTCs does not require CREB phosphorylation at Ser133 (unlike CBP), nor does it require a CREB/CBP interaction (although this interaction may occur while CRTCs bind to CREB). Nevertheless, CRTCs do not constitutively activate CREB. Instead, the CRTC/CREB interaction depends upon the subcellular localization of CRTCs. When CRTCs are phosphorylated by Salt Inducible Kinase 1 (SIK1) (in their regulatory domain) (Figure 5), CRTCs translocate to the cytoplasm, where they are sequestered (under basal conditions), due to their association with 14-3-3 scaffolding proteins [61,62].

Phosphorylated, cytoplasmic CRTCs are coincidence detectors, which respond to simultaneous increases in [Ca^2+^]_in_ and cAMP by their translocation to the nucleus (and interaction with CREB) [61,63]. The increase in [Ca^2+^]_in_ is required because Ca^2+^ activates PP2B (calcineurin), which dephosphorylates CRTCs [61,63] (Figure 5). However, calcineurin activation is generally not sufficient for CRTCs to enter the nucleus, because after their dephosphorylation by calcineurin, CRTCs are rapidly rephosphorylated, primarily by SIK1 (or other members of the AMP-activated protein kinase (AMPK) family) [64,65]. 

**Figure 6:**
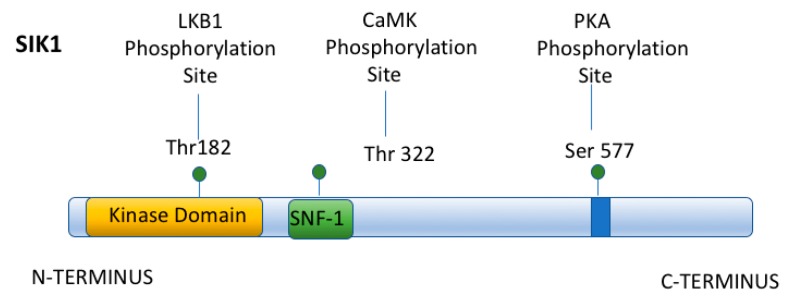
Structure of SIK1. SIK1 has an amino terminal Kinase domain that can be phosphorylated by LKB1, activating SIK1. SIK1 can also be phosphorylated at Thr322 by CaMK and activated. In contrast, SIK1 can be phosphorylated at Ser577 by PKA and inhibited.

The kinase activity of SIK1, like that of other AMPKs, is subject to regulation by phosphorylation events. While SIK1’s kinase activity is activated following phosphorylation by either Liver Kinase B1 (LKB1) at Thr182, or CaMK at Thr322, SIK1 is inhibited following its phosphorylation at Ser577 by PKA (Figure 6) [51,66]. Thus, when intracellular cAMP increases concomitantly with an increase in [Ca^2+^]_in_, CRTCs remain dephosphorylated and translocate to the nucleus, where they interact with CREB on a subset of CREB-regulated genes.

## 6. Involvement of CREB and CRTCs in Transcriptional Regulation of *atp1b1*

A number of studies indicate that the Na,K-ATPase β1 subunit gene (*atp1b1*) is one such CREB/CRTC/SIK1 regulated gene [67]. Studies concerning the effects of PGE_1_ and PGE_2_ on *atp1b1* gene regulation in dog kidney epithelial cell line MDCK [48] indicated that transcriptional upregulation of *atp1b1* depends upon the activation of Gs coupled EP1, as well as Gq coupled EP2 receptors, which activate Adenylate Cyclase (AC) and Phospholipase C (PLC) [49], respectively. Indeed, the inhibition of either PKA activation (by PKI, the PKA inhibitory peptide) or Ca^2+^/calmodulin (by the calmodulin antagonist W7) prevented the stimulatory effects of PGE_1_ and PGE_2_ on *atp1b1* transcription [49]. Thus, signaling via both cAMP and Ca^2+^ must occur in order for PGE_1_ and PGE_2_ to stimulate atp1b1 transcription, suggesting the involvement of the coincidence detectors CRTCs. 

Consistent with the involvement of CRTCs, CRTC expression vectors (encoding for either CRTC1, CRTC2, or CRTC3) stimulated transcription in transient transfection studies with pHβ1-1141 Luc (a human Na,K-ATPase β1 promoter/luciferase construct) [6,38]. Initially, it was unclear which of these CRTCs was responsible for stimulating *atp1b1* transcription, because Western analyses indicated that all three CRTCs are expressed in MDCK cells, as well as in primary cultures of rabbit RPT cells. However, the major CRTC expressed in both of these renal cell culture systems is CRTC1 [6]. In this manner, renal tubule epithelial cells resemble cells in the brain, where CRTC1 is similarly the major CRTC isoform [68]. In order to examine the requirement of CRTC1, studies were conducted with a dominant negative (DN) CRTC1 vector [6]. The results indicated that in RPT cells, the DN CRTC1 prevented the PGE_2_ stimulation of transcription observed with pHβ1-1141 Luc. Similarly, the DN CRTC1 vector interfered with hippocampal late phase long term potentiation in the brain [63]. The other two CRTC isoforms play a major role in metabolic regulation in a tissue specific manner. CRTC2 plays a major role in the liver, and in the pancreatic islets, where it promotes the expression of gluconeogenesis [51] and insulin production, respectively [69]. In adipocytes, CRTC3 plays a major role in lipid metabolism, with CRTC3 polymorphisms being associated with obesity [70]. 

## 7. Regulation of the Na,K-ATPase β1 Subunit Gene *atp1b1* in the Renal Proximal Tubule

**Figure 7:**
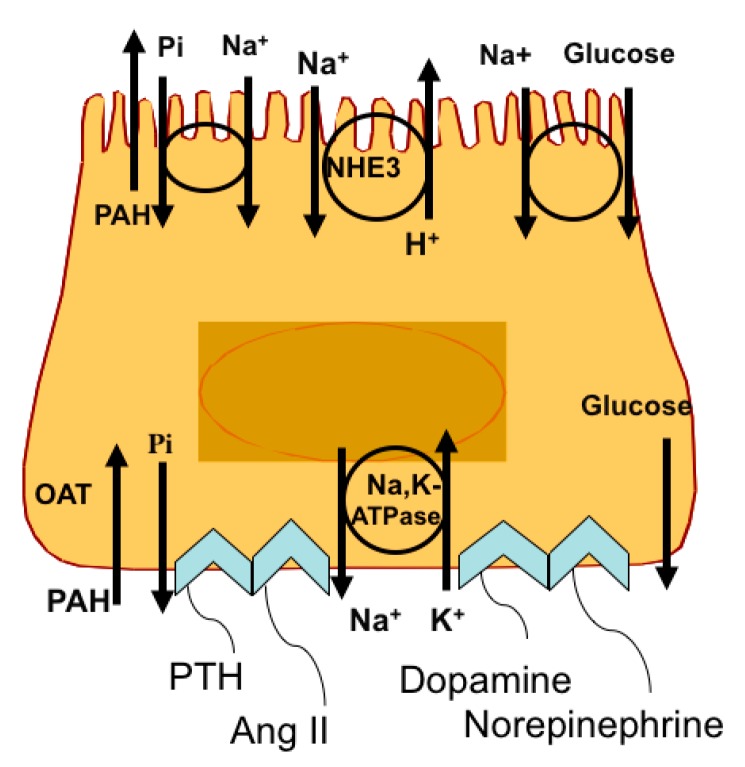
Primary RPT cells have a polarized morphology with an apical surface facing the medium in vitro and a basolateral surface facing the culture dish. The cultures possess a Na^+^/Pi cotransport system (NaPi2a), a Na^+^/H^+^ antiport system (NHE3), and a Na^+^/glucose cotransport system (SGLT2) on the apical surface, facilitating Na^+^, Pi, and glucose reabsorption. The Na^+^ gradient required for reabsorption is provided by the basolateral Na^+^,K^+^-ATPase, which is also is responsible for the efflux of Na^+^ into the interstitial space. The RPT cells also have a basolateral p-AH transport system responsible for the secretion of organic anions into the lumen of the nephron. The primary RPT cells also have receptors for PTH, Ang II, dopamine, and norepinephrine.

In order to obtain an understanding of the regulation of the RPT *atp1b1* gene by norepinephrine and dopamine, subsequent studies were conducted with primary rabbit RPT cells. Primary rabbit RPT cell cultures are particularly appropriate for such studies because these primary cultures retain a number of differentiated RPT functions [71,72] (Figure 7). Included amongst these functions are (1) apical transporters, such as the Na^+^/glucose cotransporter (SGLT2) [71], the Na^+^/Pi cotransporter (NPT2a; SLC34A1) [73,74], and the Na^+^/H^+^ antiporter (NHE3), (2) basolateral transporters such as the p-Aminohippurate (pAH) transporter (OAT1) [75], and Na,K-ATPase [76], (3) metabolism including gluconeogenesis [77,78], and (4) responses to hormones and other effectors including Parathyroid Hormone (PTH) [71], Angiotensin II (Ang II) [79], norepinephrine (α and β adrenergic receptor-mediated) [6], and dopamine (DA1 and DA2 receptor-mediated) [6]. Furthermore, the cultures are polarized [80] and form functional tubules in matrigel [81]. 

The primary rabbit RPT cell cultures have been used extensively to study the regulation of a number of transporters responsible for the transepithelial transport of a number of solutes including glucose, phosphate, and other ions [74,82,83,84]. A number of regulatory pathways have been identified. For example, arachidonic acid, phospholipase A2, and 5,6 epoxyeicosatrienoic acid, have been found to be mediators of the effects of Angiotensin II on Na^+^ transport [79,85,86]. The effects of high glucose on transport by primary RPT cells have been examined so as to understand the changes that occur in the RPT during diabetes [82,87]. The effects of toxicants have been studied extensively in primary rabbit RPT cell cultures [88,89,90], given that this nephron segment is a target of a number of drugs, oxidants, and heavy metals. Finally, primary rabbit kidney cell cultures have been the source of a number of established cell lines, including those originating from the proximal tubule [91,92,93]. In the area of kidney physiology, this is particularly important because the effects of prolonged exposures to hormones, growth factors, and other effectors can be more readily studied with renal cell cultures than with isolated tissues [91]. Admittedly, there is the potential problem of changes occurring in the expression of differentiated function in the culture situation, particularly in the case of established kidney cell lines. For this reason, it is important to check the physiological significance of in vitro studies with the in vivo situation. 

Primary RPT cell cultures have been used to study the signal transduction pathways underlying the chronic regulation of Na,K-ATPase, including the chronic effects of catecholamines. 

Although the acute effects of catecholamines on Na^+^ reabsorption by the RPT have been extensively studied, the chronic effects of catecholamines on Na^+^ reabsorption by the RPT are not well understood. Nevertheless, there is strong evidence for chronic control. Normally, the renal sympathetic nervous system is activated when Na^+^ intake declines, resulting in the production of norepinephrine by renal nerves. Chronic increases in renal sympathetic nerve activity are well documented [94]. The resulting hypertension has been attributed at least in part to increased RPT reabsorption of Na^+^. Consistent with this hypothesis, chronic norepinephrine infusion in rats has been observed to increase the levels of RPT ion transporters [95]. Further evidence for chronic regulation by norepinephrine has come from the alterations in renal Na^+^ excretion which are observed following renal denervation, or the stimulation of renal nerves in anesthetized animals [94]. Indeed, renal denervation has been employed as a means to reduce blood pressure, presumably by reducing Na^+^ reabsorption by the RPT [96]. 

Chronic Na^+^ loading results in increased renal dopamine excretion and natriuresis (as observed with the acute response), although the underlying mechanisms are different [97,98]. While the inhibition of Na^+^ reabsorption during the acute response involves changes in the phosphorylation of a number of RPT Na^+^ transporters (including NHE3 as well as Na,K-ATPase), and altered trafficking, chronic regulation has been proposed to involve changes in the expression of RPT Na^+^ transporters, which may occur at the transcriptional as well as the translational level [99]. When these mechanisms are blocked by either the pharmacological or genetic disruption of dopamine receptors (including DA1 and DA2), natriuresis and volume-dependent hypertension (which normally occur) are impaired [100]. Similarly, when the production of dopamine by the RPT is blocked in transgenic mice (with a KO of the RPT aromatic amino acid decarboxylase enzyme), the expression of mRNAs for RPT Na^+^ transporters is increased, and the mice develop hypertension on a high salt diet. However, the mechanisms responsible for the increased expression of Na^+^ transporters in the KO RPT are not understood.

## 8. Regulation of Na,K-ATPase Gene Expression by Adrenergic Agonists

For this reason, the effects of adrenergic and dopaminergic agonists on the expression of the Na,K-ATPase β subunit gene (*atp1b1*) in primary RPT cells were examined. Transient transfection studies with the human *atp1b1* promoter/Luciferase construct pHβ1-1141Luc indicated that norepinephrine stimulated the transcription more than two-fold. This stimulatory effect was inhibited by the α adrenergic antagonists prazocin and yohimbe [6]. The involvement of β adrenergic receptors was indicated by the stimulatory effect of β adrenergic agonists (including albuterol and metoprolol) on *atp1b1* transcription (in addition to stimulatory effects of the α adrenergic agonists phenylephrine and guanabenz). Therefore, both a β adrenergic mediated increase in cAMP and an α adrenergic increase in [Ca^2+^]_in_ would be expected in primary RPT cells treated with norepinephrine.

Thus, the treatment of RPT cells with adrenergic agonists would be expected to cause the activation of PP2B (by Ca^2+^/calmodulin), the inhibition of SIK1 (due to its phosphorylation by PKA), and ultimately, the nuclear translocation of CRTCs, as observed in other tissues, including rat skeletal muscle [101] and in rat pinealocytes [102]. Once in the nucleus, CRTCs would be expected to interact with CREB and stimulate transcription (as shown in Figure 5). The results obtained with primary RPT cells are consistent with this hypothesis, including the inhibition of the norepinephrine stimulation of *atp1b1* transcription by a DN CRTC1, as well as CREB R314A, which lacks the CRTC1 binding site [6]. Presumably, CREB R314A is expressed in excess, and thus binds to the human atp1b1 promoter, rather than endogenous CREB, thereby preventing a normal CREB/CRTC interaction from being established in this promoter.

The results obtained with SIK1 K56M are also consistent with the involvement of CRTC1 as a mediator of the stimulatory effects of norepinephrine. In the absence of norepinephrine, the basal level of *atp1b1* transcription increased three-fold in primary RPT cells cotransfected with SIK1 K56M. The increased basal level of *atp1b1* transcription can be attributed to the inability of endogenous SIK1 to phosphorylate CRTC1 in this condition (given the presence of excess SIK1 K56M). As a consequence, unphosphorylated CRTC1 accumulates in the nucleus, and associates with CREB independently of changes in intracellular Ca^2+^ and cAMP. The results obtained with norepinephrine-treated primary cultures cotransfected with SIK1 K56M are also consistent with the hypothesis that the CRTC1 activates CREB on *atp1b1.* In the presence of SIK1 K56M, norepinephrine did not increase *atp1b1* transcription above the level observed in untreated primary RPT cells cotransfected with this kinase dead SIK1. This latter observation can be explained if a maximal CREB/CRTC interaction had already occurred in these cells in the absence of an increase in Ca^2+^ and cAMP. 

## 9. Regulation of Na,K-ATPase Gene Expression by Dopamine

The effect of dopamine on *atp1b1* transcription was also studied, simulating the conditions that elicit the acute inhibitory response to dopamine in vivo. In vivo, there is a facultative dopamine-mediated system that is activated in RPT cells following an Na^+^ load [30]. Dopamine is produced by RPT cells using circulating L-DOPA as a substrate. However, in addition to the requirement for dopamine production, the transport response of RPT cells to dopamine also requires an increase in [Na^+^]_in_ (which occurs in vivo as a consequence of an increased level of Na^+^ in the lumen of the nephron). In order to obtain an inhibitory effect of dopamine on Na,K-ATPase in vitro (in OK cells), a similar increase in [Na^+^]_in_ was obtained using monensin [103]. The increase in [Na^+^]_in_ which occurs in the presence of monensin (in the absence of an external effector) results in an acute increase in Na,K-ATPase activity in OK cells, which can be attributed to SIK1 activation [30]. Similarly, the chronic treatment of primary RPT cells with monensin results in increased *atp1b1* transcription [6]. Importantly, the stimulatory effect of monensin on *atp1b1* transcription was inhibited by dopamine. Both the monensin stimulation and the dopamine inhibition were prevented by kinase dead SIK1, SIK1 K56M [6], consistent with the involvement of SIK1 [6].

Of particular interest are the results obtained in transient transfection studies when dopamine is added in the absence of an increase in [Na^+^]_in_. Dopamine is stimulatory to *atp1b1* transcription under these conditions. The activation of both DA1 and DA2 receptors by dopamine in the primary RPT cells presumably results in an increase in cAMP and [Ca^2+^]_in_. These conditions favor CREB activation, the nuclear translocation of CRTCs, and increased *atp1b1* transcription. Such a stimulatory effect of dopamine on *atp1b1* transcription would not be expected in vivo in the RPT, because dopamine is only produced by RPT cells when Na^+^ levels increase in the lumen of the proximal tubule (due to a high Na^+^ intake). 

Following an increase in luminal Na^+^, [Na^+^]_in_ increases, followed by an increase in [Ca^2+^]_in_, and the activation of CaMK1. Thus, when dopamine is produced in vivo, SIK1 has already been activated in the RPT due to its phosphorylation at Thr322 by CaMK1. In the absence of dopamine, SIK1 activation by CaMK1 ultimately results in increased transcription by MEF2 and/or NFAT, due to the class IIa HDAC Kinase activity of SIK1, as described below. However, dopamine can prevent this effect by stimulating the phosphorylation of SIK1 at Ser577 (via PKA), thus inhibiting SIK1’s kinase activity.

## 10. Overall Role of CRTCs and SIKs in Regulating Sodium Homeostasis and Metabolism

Overall, these investigations thus suggest that CRTC1 and SIK1 play an important role in Na^+^ homeostasis in the kidney, and in this manner contribute to the regulatory role played by SIK1 in sodium homeostasis in other organs, including the adrenal. Indeed, SIK1 was originally cloned from the adrenals of rats fed a high salt diet (followed by the cloning of two other SIK isoforms, including SIK2 in adipocytes, as well the ubiquitous SIK3) [104,105]. After the induction steroidogenesis in the adrenal, the SIK1 gene (which is transcriptionally regulated by CREB) is upregulated. As a consequence, CRTCs are phosphorylated by SIK1, and transcriptional regulation by CRTCs is inactivated (creating a negative feedback loop) [106]. These effects of SIK1 on the adrenal constitutes only a part of the role played by SIK1 in the hypothalamic-pituitary-adrenal axis. Indeed, SIK1 also represses Corticotropin-Releasing Hormone (CRH) transcription in hypothalamic neurons [107], and in this manner prevents the stimulatory effect of CRH on the synthesis of ACTH by the pituitary. 

The physiologic role of the CRTC/SIK pathway is not restricted to sodium homeostasis. Indeed, since their discovery, CRTCs have been studied most extensively with regards to their role in metabolic regulation [51]. CRTCs have been studied in a number of peripheral, insulin sensitive tissues (including the liver, muscle, adipose tissue, and pancreas) [51], as well as in the brain [108]. For example, in the liver, the transcription of genes encoding for proteins involved in gluconeogenesis (including Phosphoenolpyruvate Carboxykinase (PEPCK) and glucose-6-phosphatase) increases during short term fasting, in part due to a CRTC2/CREB interaction in the PEPCK promoter [109]. CRTCs also play an important role in metabolic regulation by the brain, which controls the secretion of hormones and other neuropeptides. For example, CRTC1 (the major brain CRTC) promotes the expression (and release) of Calcitonin Gene-Regulated Peptide (CGRP) by the dorsal root ganglion neurons, which targets insulin secretion (pancreas) [108]. Similarly, catecholamines released by sympathetic neurons activate CRTC2/3 in skeletal muscle, so as to increase the density of myofibers, in addition to increasing glycogen and triglyceride stores (ultimately increasing endurance during exercise) [108]. 

## 11. Physiologic Consequences of the HDAC Kinase Activity of SIK1

Recent studies indicate that SIK1 controls transcription not only by phosphorylating CRTCs, but in addition by yet another mechanism that ultimately affects Na^+^ homeostasis. This latter mechanism of transcriptional regulation by SIK1 was initially revealed from studies of transgenic mice that express a DN CREB in skeletal myocytes (M-ACREB mice) [110]. Myogenic gene expression was reduced in the skeletal myocytes in the M-ACREB mice. However, it was hard to understand how CREB exerted its control, because the myogenic genes did not have CREB binding sites. However, studies indicated that the dystrophy in the M-ACREB mice could be explained specifically by the reduced expression of SIK1 in the M-ACREB mice. Normally CREB upregulates SIK1 gene expression when cAMP levels increase, and this regulation was lost in the myocytes of M-ACREB mice. Berdeaux et al. [110] discovered that the reduced expression of SIK1 was ultimately responsible for the reduced expression of myogenic genes.

The transcription of most myogenic genes directly depends upon the binding of Myocyte-specific-enhancer-factor-2 (MEF2) transcription factors to core motifs in the promoter region (which have a sequence similar to the 5′-CT(A/t)(a/t)AAATAG-3′ consensus sequence). Despite the binding of MEF2 to such core motifs, the transcriptional activity of MEF2 is nevertheless repressed by the binding of HDAC5 (or other class IIa HDACs) to MEF2 proteins present in promoter regions. However, following the phosphorylation of class IIa HDACs, these HDACs dissociate from MEF2, resulting in the derepression of myogenic genes. SIK1 was identified as being the kinase responsible for the phosphorylation of the class IIa HDACs which were present on myogenic genes.

The role of SIK1 as a class IIa HDAC Kinase is also important in the regulation of genes in other tissues. Popov et al. [111] conducted studies with an atrial derived cell line HL-1, which expresses the same hypertrophic markers as cardiomyocytes. They observed that after [Na^+^]_in_ was increased in HL-1 cells (using monensin), SIK1 was activated, which resulted in HDAC5 phosphorylation, and, as a consequence, increased transcriptional activity of MEF2, as well as Nuclear Factor of Active T cell (NFAT). Increased expression of a number of hypertrophic genes was observed, including cardiac α-myosin heavy chain (α-MHC) and brain natriuretic peptide (BNP). Subsequently, Popov et al. [112] observed that the knockout of another SIK isoform, SIK2, prevented the development of the left ventricle hypertrophy that normally occurs in response to a high salt diet, possibly as a consequence of class IIa HDAC phosphorylation.

**Figure 8:**
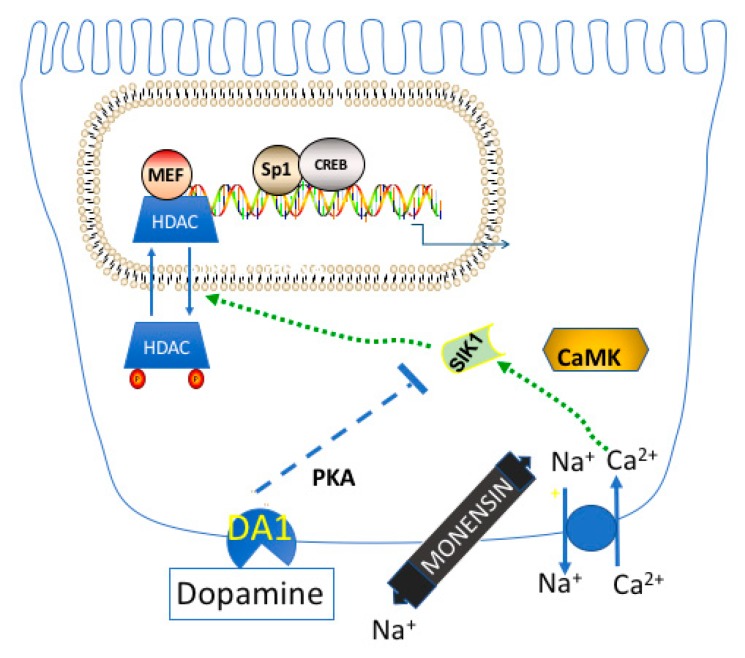
Monensin treatment results in the activation of CaMK, which phosphorylates and activates SIK1. As a consequence, class IIa HDACs are phosphorylated by SIK1, and translocate to the cytoplasm, resulting in the derepression of transcription by MEF2 and NFAT transcription factors.

SIK1 activation in the RPT similarly would be expected to result in an increase in the expression of genes regulated by MEF2 (and NFAT), including Na,K-ATPase. The report of Sjöstrom et al. [30] indicates that a monensin-induced increase in [Na^+^]_in_ results in the activation of SIK1 in the OK cell line, which possesses a number of RPT functions. SIK1 is similarly activated in primary RPT cell cultures, presumably resulting in the phosphorylation of class IIa HDACs, and, as a consequence, the increased expression of MEF2/NFAT regulated genes, including *atp1b1*. The hypothesis that the stimulatory effect of monensin on *atp1b1* transcription depends upon the phosphorylation of class IIa HDACs can be readily evaluated using a phosphorylation defective HDAC5 S259A/S498A expression vector. According to this hypothesis, the monensin stimulation would be lost in primary RPT cells transfected with phosphorylation defective HDAC5 S259A/S498A, because SIK1 can no longer phosphorylate HDAC5 at S259 and S498 (an event which causes HDAC5 to lose it binding affinity for MEF2 and/or NFAT) [111] . Therefore, HDAC5 (and other class IIa HDACs) constitutively binds to MEF2 and/or NFAT located on promoter regions of responsive genes. As a consequence, the expression of MEF2/NFAT target genes is repressed. In the presence of dopamine, the monensin stimulation is lost, presumably due to the activation of PKA in dopamine-treated RPT cells, and as a consequence, the inhibition of SIK1 (and its class IIa HDAC Kinase activity) (Figure 8).

## 12. Conclusions

The studies described above indicate that renal effectors regulate Na,K-ATPase in the RPT by two distinct, but inter-related, signaling pathways. On one hand, simultaneous increases in intracellular cAMP and Ca^2+^ are sensed by CRTCs, which translocate to the nucleus, where they interact with CREB, thereby increasing the transcription of a subset of CREB-regulated genes, including the Na,K-ATPase β1 subunit gene *atp1b1*. On the other hand, an increase in [Ca^2+^]_in_ results in CaMKI activation, and the subsequent activation of SIK1. Activated SIK1 phosphorylates class IIa HDACs, which lose their affinity for genomic MEF2/NFAT, thereby activating the expression of MEF2/NFAT regulated genes. The CaMKI activation which initiates these events is likely a consequence of an increase in [Na^+^]_in_ in RPT cells in vivo, caused by an increase in Na^+^ in the lumen of the RPT.

A number of questions arise from the studies described here concerning CRTCs and SIK1, which can be addressed by new directions of research. Of particular interest in these regards is the overall role of CRTCs and SIK1 in the RPT in blood pressure regulation. The role of CRTCs and SIK1 in regulation of the RPT Na,K-ATPase has been defined. However, Na,K-ATPase is only one of the transporters responsible for Na^+^ reabsorption by the RPT. Luminal Na^+^ initially enters RPT cells via apical transporters, including the Na^+^/H^+^ antiport system NHE3, as well as Na^+^ cotransporters, including the Na^+^/Pi cotransporter NaPi2a. The possibility of coordinate transcriptional control of these transporters with Na,K-ATPase needs to be evaluated (including the involvement of CRTC1 and SIK1). Like Na,K-ATPase, both NHE3 and NaPi2a are subject to acute regulation by dopamine and adrenergic agonists [113,114,115,116,117]. Little is known about the transcriptional regulation of the genes encoding for NHE3 and NaPi2a (SLC9A3 and SLC34A1, respectively). However, CREB binding sites have been identified in the promoter region of each of these two genes [118,119]. Thus, these genes are indeed candidates for being subject to transcriptional control by CRTCs and SIK1. 

An important issue in these regards is the nature of the role of the RPT in blood pressure regulation, including the involvement of SIK1, as well as CRTC1. Studies have been conducted concerning the effect of a global SIK1 knockout (sik1^−/−^) on blood pressure regulation. While global sik1^−/−^ mice exhibit a similar blood pressure to sik1^+/+^ mice under normal salt conditions, during chronic high salt intake (1% NaCl), the mice exhibit high systolic blood pressure and signs of cardiac hypertrophy [120]. Although the RPT has not yet been studied, urinary levels of Na^+^, K^+^, and Cl^-^ are increased in sik1^−/−^ mice maintained on both a normal and a high salt diet [120]. Future studies are needed to examine blood pressure regulation and the effect of high salt intake in mice with a sik1 knockout directed specifically to the RPT. SIK1 also interacts with Na,K-ATPase itself in the RPT. Thus, the effect of an RPT directed knockout of CRTC1 is also important to study, in order to gain an understanding of the specific role of SIK1, as well as CRTC1, in transcriptional control of ion transporters in the RPT and blood pressure regulation. Of course, in the case of an RPT directed CRTC1 knockout, concerns could be raised about alterations in the renal circadian clock, which also involves CRTC1. Circadian clock proteins, including Period (per) proteins, are transcriptionally active in the kidney, and NHE3 has been identified as being a CLOCK-controlled gene [121]. Moreover, the CLOCK has been shown to contribute to blood pressure regulation [122] .

Previous studies have indicated that partial inhibition of Na,K-ATPase by ouabain results in an increase in [Ca^2+^]_in_, due to the activation of a Na^+^/Ca^2+^ exchange system by the increase in [Na^+^]_in_ (which occurs as a consequence of the partial inhibition of Na,K-ATPase by ouabain). At low ouabain concentrations, the signaling function of Na,K-ATPase is activated due to the interaction of Na,K-ATPase with Src and the transactivation of the EGF receptor [5]. The activation of the Ras/MAPK signaling cascade is also stimulated by an increase in Ca^2+^ and in CaMK activity. More recently, the Na,K-ATPase α1 subunit has been observed to interact with the Inositol 1,4,5-Trisphosphate Receptor (IP3R), an interaction that may be required for the release of Ca^2+^ from the ER following the interaction of extracellular effectors such as Ang II and EGF with their receptors. Of particular interest, the same caveolar Na,K-ATPase that interacts with the IP3R is part of the multiprotein complex that contains Src and the EGFR. Chen et al. [123] have proposed that the interaction between the Na,K-ATPase and the IP3R keeps the IP3R in the proximity of local Gq-coupled GPCRs, so as to facilitate Ca^2+^ release following the activation of these GPCRs. In the RPT, the proximity of the IP3R to such GPCRs would be extremely important in facilitating Ca^2+^ release following the interaction of norepinephrine and Ang II with α adrenergic and AT1 receptors, respectively. It is unclear whether the SIK1 associated with Na,K-ATPase is also present within the caveolae. However, if this is indeed the case, renal effectors would very efficiently activate SIK1 (as well as its HDAC kinase activity), and in this manner, contribute to the overall effects of caveolar Na,K-ATPase on gene expression.
